# Consumer reviews analysis on cycling pants in online shopping malls using text mining

**DOI:** 10.1186/s40691-021-00264-7

**Published:** 2021-10-15

**Authors:** Chunjeong Kim, Youngjoo Na

**Affiliations:** 1 Researcher, Institute of Symbiotic Life-TECH, Yonsei University, Seoul, 03722 Republic of Korea; 2 Professor, Department of Fashion Design and Textiles, Inha University, 100 Inha-ro, Michuhol-gu, Incheon, 22212 Republic of Korea

**Keywords:** Cycling pants, Bib shorts, Online shopping mall, Consumer reviews, Text mining

## Abstract

This study was investigated trends and consumer awareness on cycling pants by analyzing the reviews on bib shorts, bib tights, shorts, and tights in online shopping malls using text mining. The reviews and product information on cycling pants from Jan. 2017 to the first half of 2020 were crawled, and a total of 7241 cases were analyzed. The keywords of cycling pants were extracted using a Korean morphological analyzer (KoNLP), calculated to the term-document matrix, and then converted into a co-occurrence matrix. The number of reviews of cycling pants increased by 39% per year, and especially in the first half of 2020, the number of reviews has doubled over compared to the first half of last year. Bib shorts accounted for more than 50% of the number of reviews of cycling pants and received the highest rating, making them the most preferred. Positive reviews on cycling pants appeared 15 times over than that of negative reviews, and most of the cycling pants were evaluated positively. Size and cost-effective appeared as the important keywords both in positive and negative reviews. However, it was found that consumers have a difficult time choosing the size not only in the negative but also in the positive reviews. Pad was the keyword that appeared the most in negative reviews, and it was the most dissatisfied factor in the cycling pants. Therefore, in an internet shopping mall, it is necessary to provide intuitive and accurate information that is easy for consumers to understand about information on the size and pad of the cycle pants.

## Introduction

With increasing awareness of health and fitness worldwide, cycling is emerging to solve health problems such as hypertension and obesity. Also, as bicycles are used as sustainable, eco-friendly transportation in urban areas, the global cycle wear market is expected to grow by an average of 7.3% per year by 2024 (Global cycling Wear Market, 2020). Although the domestic bicycle penetration rate in Korea is still low compared to Europe (Statistics Korea, 2017), the domestic bicycle industry is expected to continue to grow due to the spread of well-being trends and the government’s measures to reduce fine dust. Kim and Yi (2018) reported that the frequency of words related to cycle wears increased annually in the internet bicycle café; this indicates that the demand for professional cycle wear increases when general consumers ride bicycles (Kim & Choi, 2017).

In recent years, the number of users who purchase products using online shopping malls has increased. The transaction volume of clothing in online shopping, including sportswear, has increased by 20% compared to the same month last year in Korea (Statistics Korea, 2020). In this way, online reviews are one of the important factors affecting purchasing a product not only when purchasing online where you cannot experience the product directly, but also when purchasing a product offline where you can directly experience them (Goldsmith & Horowitz, 2006; Zhang et al., 2014). Cycling has been receiving attention recently, and cycling wear is an important factor for cyclists regardless of long-distance or short-distance cycling. Especially, cycling pants that are exclusively for cycling wear are becoming essential purchase items because of their technical functionality when cycling (Downer & Cassidy, 2011). Cycling pants are a type of compression wear that requires various functionalities in textiles, and various domestic and foreign brands have released many products. Nevertheless, their product information is insufficient for consumers to purchase. As such, cycling pants’ performance is more important than fashion, but consumers feel that there is insufficient information on the product they want to purchase because it is difficult to evaluate the performance. Cyclists use online comments or reviews about cycling pants to get product information when purchasing them. However, with the recent rapid increase in reviews, consumers have to spend a lot of time reading all the comments and reviews to grasp the actual and objective information. It is becoming difficult to judge the product’s information. Besides, there is a lack of evidence to judge the accuracy of these numerous reviews. Looking at the Internet usage rate by country, Korea is 95.9%, the United States is 87.3%, Japan is 84.6%, and France is 82%, and on average, 90% of all individuals are using the Internet, and their usage exceeds the OECD (KOSIS, 2020; OECD, 2017), Although Korea has an excellent environment for consumer analysis related to Internet users, there are few studies on consumer analysis, especially cycling wear.

Among the psychological evaluation methods used to analyze consumers' sensibilities or desires, the most frequently used survey method is simple and convenient. However, their question is designed in advance, it is difficult to reflect various experiences of consumers (Coughlan, 2009), and there is the possibility of bias or distortion in the response. On the other hand, with the recent development of the Internet, consumers voluntarily post product-related reviews online and share consumer feelings and discomforts in real-time, which quickly allows them to grasp the honest consumer’s emotions. For this reason, the importance and essential role of reviews have been strongly emphasized year and year (Felbermayr & Nanopoulos, 2016; Kawaf & Istanbulluoglu, 2019). In particular, high-performance products such as cycling pants are both search products and experience products, so that reviews of products have written by users after using the product will be a greater impact when consumers purchase the products.

Therefore, this study crawled the reviews and product information of cycling pants on online shopping malls and analyzed their trends and sensibilities using text mining. In addition, by dividing the review of cycling pants into positive and negative, they were analyzed consumer awareness and examined the utility of using these reviews when consumers purchase products. These can have utilized as basic data for the recommendation system related to cycling wear in the future.

## Literature review

### Text mining and sentiment analysis

Recently, the new formats of the text, different from the existing ones, are increasing exponentially on social media such as Facebook, KakaoTalk, Twitter, blogs, and online shopping malls. Text mining is a research technique that collects, processes, analyzes, and summarizes these text-type unstructured data in text format based on natural language processing technology and can extract useful information that could not be found before (Back, 2017; Feldman, 2007). Like word of mouth that consumers hear offline, online reviews can help consumers get more reliable information about their product. Consumers can reduce the risk of purchasing and lower prices through reviews from other consumers. They found that they trusted other consumers’ reviews more than advertisements and made this an important purchasing decision criterion (Goldsmith & Horowitz, 2006). Consumers are likely to show a favorable response to the product by increasing the number of online reviews (Dhar & Chang, 2009) because the number of online reviews has increased consumer awareness of the product and implied the product is of interest to consumers.

Researches that analyze consumer perception of online social data about fashion products using text mining are as follows. In outdoor clothing, they were extracted positive related words such as ‘various,’ ‘excellent,’ ‘practical,’ ‘comfortable’ and negative related words such as ‘expensive,’ ‘uncomfortable,’ ‘fake,’ and ‘tough’ (Jung & Oh, 2016). Positive keywords in luxury brands appeared ‘various,’ ‘famous,’ 'excellent,’ ‘perfect,’ and ‘luxurious’ and negative keywords were ‘expensive,’ ‘price,’ ‘old,’ ‘bland,’ ‘tough,’ and ‘fake’ (Kim & Kim, 2016). Topic modeling of online posts on striped shirts resulted in top-level associations such as ‘pattern,’ ‘suits,’ and ‘coordinates’ (An & Park, 2017). In swimsuits, ‘size,’ ‘design,’ and ‘price’ appeared as upper words, indicating that consumers valued the internal image rather than the external image of swimsuits (Lee et al., 2017). Professional cycling wear demands are increasing in an internet cafe, and the important factors were ‘price,’ ‘size,’ and ‘brand’ (Kim & Yi, 2018). Like these, sensibility analysis using text mining (Na, 2011) has been investigated for various fashion products, but there are few analyzed about sensibilities in online reviews on cycling pants.

### Cycling pants

**Fig. 1:**
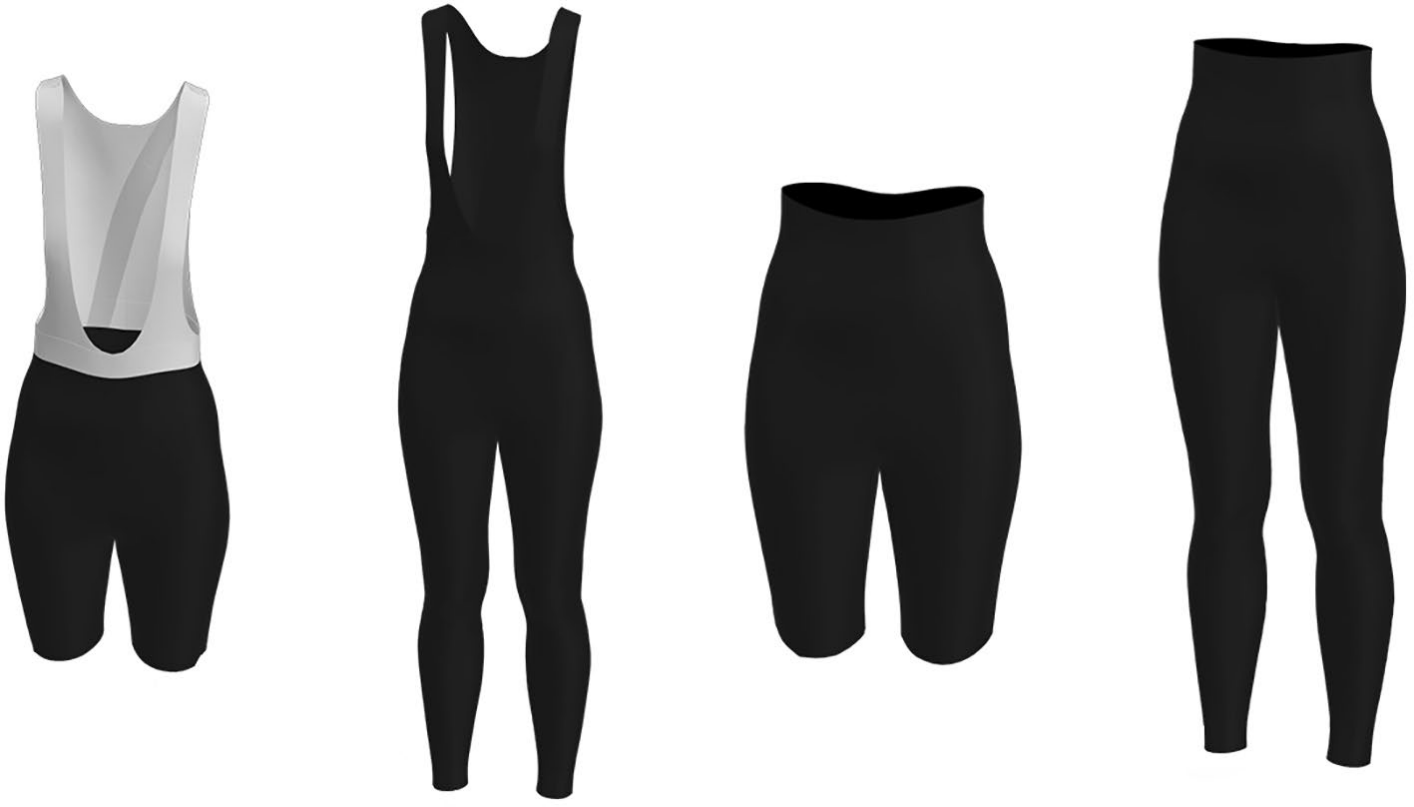
Types of cycling pants (Reprinted from https://bike.shimano.com)

The cycling pants, as shown in Fig. 1, are generally composed of bib shorts and bib tights in the suspenders style with shoulder straps and shorts and tights in the leggings style without shoulder straps. It is made of elastic material and sticks tightly to the body. In addition, the pad is attached to protect the perineal and hipbone region and reduce saddle pain that occurs when cycling for a long time. The pad is generally formed in a wider rear area of the saddle and has a saddle shape that gradually narrows toward the center (Maier, 2011, 2016). It is important for pads to make accurate contact with the saddle when pedaling to reduce saddle pain. Its back is designed to slightly raised to be suitable for skin elongation according to the streamlined posture when riding a bicycle, and a spandex gripper is used on the hem to prevent the problem that the ends of the pants roll up when pedaling (Choi et al., 2001).

The important properties of textiles required for cycling pants are quick sweat absorbency and quick-drying to release heat and sweat generated inside the body, and also excellent elasticity, and the elastic recovery rate for tightening leg muscles and increasing athletic performance. Also, functions such as excellent abrasion strength, washing durability, anti-static, antibacterial, dimensional stability, UV protection, reduced air resistance, etc., are required (Venkatraman et al., 2013). Bibs shorts and bib tights are often used by riders because the shoulder straps that run from front to back through the trousers and shoulders do not easily dislodge from the violent movements that may occur in riding. However, the shoulder strap may become loose and fall off the rider's shoulder, or conversely, the shoulder strap may be pressed too tightly against the rider's shoulder (Okajima, 2006).

Cycling pants are becoming a must-have item when riding a bicycle because it tightens the muscles of the body to prevent muscle relaxation as well as the pad attached to the bottoms prevent saddle pain.

## Method

### Crawling web data on cycling pants in the online shopping mall

The data were targeted cycling pants with pads such as bib shorts, bib tights, shorts, and tights at Naver shopping mall (https://shopping.naver.com) in Korea. It is possible to collect consumer reviews at one website for these products in the various domestic online shopping malls because Naver shopping supports a product search service through several open markets such as G Market, Auction, 11th Street, Interpark, home shopping. Web crawling used Python ver.3 to collect product names, brand, user reviews, ratings, and review dates for each product from January 1, 2017 to Jun 30, 2020. There were 7.257 cases of data collected, and a total of 7241 cases were used for analysis except for 16 meaningless data, such as having only product names in the review area. The crawled cycling pants were 42 products with ten brands, including Sant**, Ard**, NS*, M**, and San**. Table 1 shows the number of brands, products, and reviews for each clothing type for the cyclewear bottoms collected by crawling.

**Table 1:** Information of crawling data on cycling pants

Category	Number of brands (n)	Number of products (n)	Range of average rating of each item^a^	Number of reviews (n)
Bib shorts	5	21	4.5–4.9	4182
Bib tights	2	2	4.5–4.7	123
Shorts	6	10	4.5–5.0	1527
Tights	5	9	4.4–5.0	1409

^a^It was converted from one to five according to a star rating of the items

### Text mining and data analysis

The collected data was analyzed using KoNLP Packages, a Korean morpheme analyzer, using R ver. 4.0.2. In the morpheme analysis, a dictionary was used by adding a user dictionary consisting of terms related to cyclewear in addition to the Sejong dictionary, and nouns, adjectives, and verbs were extracted by removing stopwords that have no meaning in interpretation, such as punctuation marks, numbers, conjunctions, and postposition. Pre-processing was performed on similar words and synonyms in the extracted corpus, and the term-document matrix was calculated. If the same words such as “good, good, good, good, good” were repeated in one document, the frequency of occurrence of the string could be overestimated, so the weightBin function was used to count duplicate strings only once. In addition, a co-occurrence matrix for network analysis was calculated. The word cloud was visualized using the ggplot2 package, and the network analysis was visualized using Gephi ver. 0.9.2. The keywords in the text mining were reviewed and translated into English by two doctoral apparel experts.

Moreover, frequency analysis, chi-square test (χ^2^), and one-way analysis of variance were conducted to examine the difference in the number of reviews and ratings for the type of cycling pants using SPSS ver. 25. It also investigated and compared the searching trends for cycling pants through Google trend analysis.

## Results and discussion

### Trends in cycling pants

**Fig. 2:**
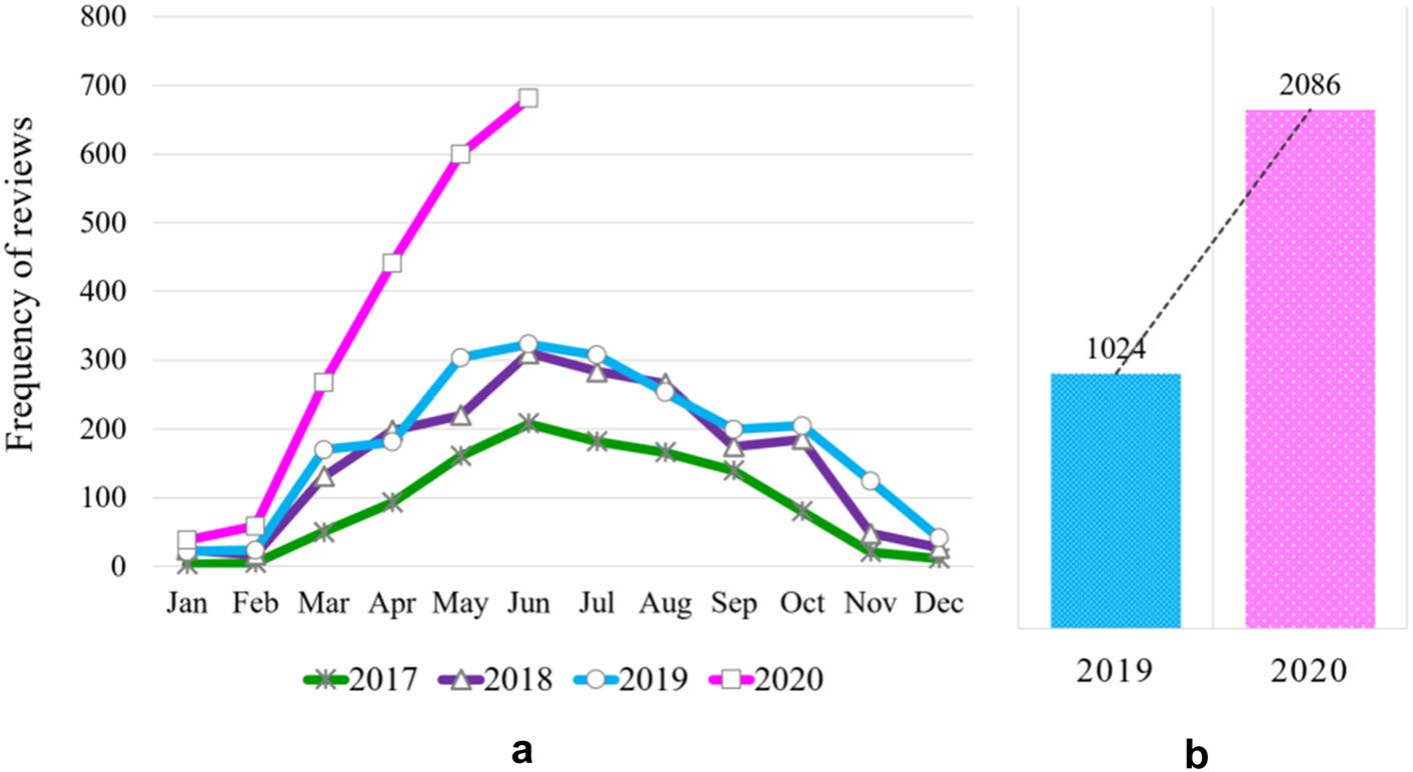
Review frequencies for cycling pants; **a** monthly reviews from 2017 to 2020, **b** total number of reviews from January to June 2019 and 2020

Figure 2a shows the review frequency for cycling pants by month and year from 2017 to 2020. It showed 1119 cases in 2017, 1,882 cases in 2018, and 2154 cases in 2019, and an annual average increase of 39% for three years, and the frequency of the first half of 2020 especially increased very rapidly. According to the monthly trends, there were fewer than 50 reviews during the winter from December to February, and it began to increase in March and showed the highest number of reviews for cycling pants since July. In particular, in 2020, the number of reviews increased rapidly from February to June, showing a different pattern from the previous one.

To analyze this in more detail, the total number of reviews from January to June, both 2019 and 2020, is shown in Fig. 2b. The number of consumer reviews was 1024 cases in the first half of 2019 and 2,086 cases in the first half of 2020 so that that of 2020 this year were doubled over compared to the same period last year. It means to indicate a sharp increase in demand for them. As this, online shopping reviews are written by consumers after purchasing products; therefore, by analyzing the frequency of reviews of products, it is possible to infer purchase trends by year and month, like as reported that the increase in the frequency of product keywords online showed a correlation with the increase in sales (Vosen & Schmidt, 2011).

**Fig. 3:**
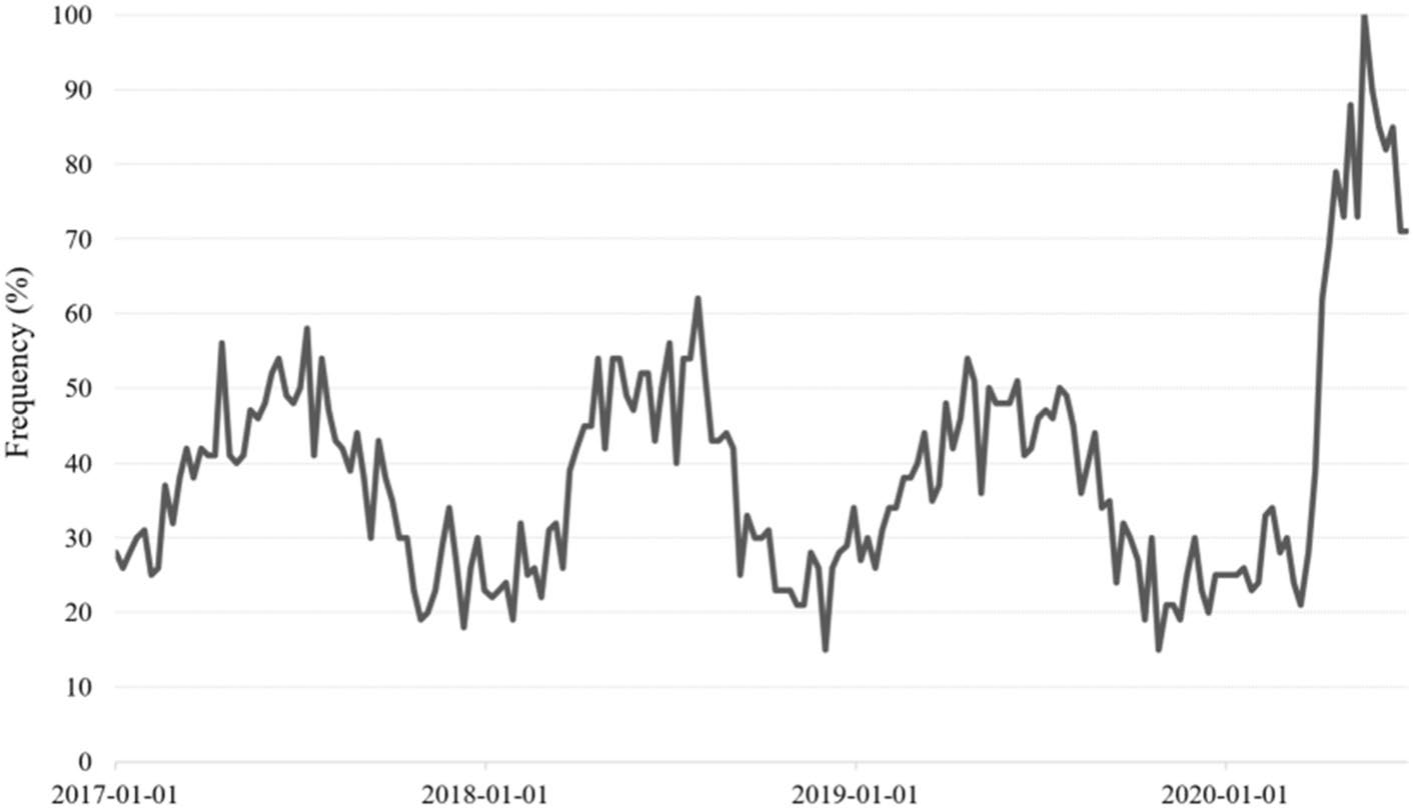
Online search trends for bib shorts from 2017.1 to 2020.6. It was analyzed by google trends analysis at https://trends.google.com. The frequency value appears as 100 for the search term with the highest frequency, 50 for the search term with half the frequency, and 0 if there is not enough data for the search term

As a result of analyzing the change of people’s interest in bib shorts through Google trend analysis (Fig. 3), it was found that a similar pattern was repeated every year from 2017 to 2019, while the frequency of bib shorts was increased sharply since March 2020. By showing the same result as the trend of the cycling pants review above, it could be confirmed once again that interest in cycling pants leads to purchase. The main factor of these results is believed to be closely related to the increase in bicycle sales because it was preferred bicycle instead of public transportation as short-distance transportation due to the influence of COVID-19 (Jung & Yang, 2020).

### Satisfaction according to types of cycling pants

As for the number of the reviews (Table 2), bib shorts accounted for 4182 cases, 56% of the total number, shorts 1527 cases, 22%, tights 1409 cases, 19%, and bib tights 123 cases accounting for 1%. Among these, bib shorts accounted for more than 50% of them are showing the most purchased.

**Table 2:** Differences of the rating of reviews on cycling pants

Cycling pants	N	Rating		F	Post-hoc^a^
		Mean	S.D		
Bib shorts (a)	4182	4.75	0.59	14.63^***^	b, c, d < a
Bib tights (b)	123	4.59	0.69		
Shorts (c)	1527	4.67	0.67		
Tights (d)	1409	4.65	0.63		

^***^ p < 0.001

^a^Post-hoc test was performed using the Scheffe’s method

A one-way analysis of variance was performed to find out whether there was a difference in the rating according to the cycling pants (Table 2), and it was found a significant difference (F = 14.63, p < 0.001). As a result of Scheffe’s post-hoc test, the number of reviews of bib shorts showed statistically significant differences compared to bib tights, shorts, and tights, but there were no significant differences among others. Although online bicycle cafes had shown more frequency of shorts and tights than that of bib shorts and bib tights (Kim & Yi, 2018), but these days, as sports apparel products have become more specialized and consumers’ awareness has increased, bib shorts have become more preferred.

**Fig. 4:**
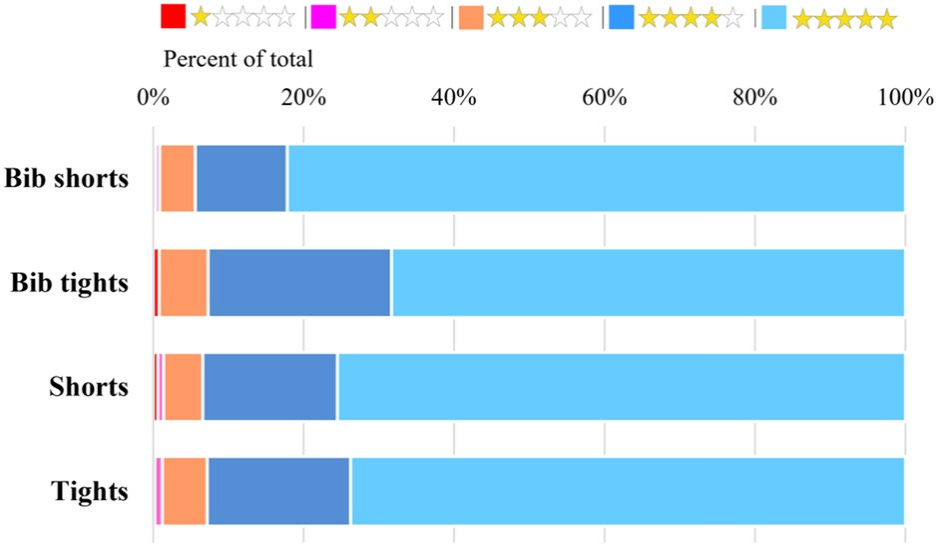
Star rating of cycling pants

Figure 4 shows the rating for each type of cycling pants as a percentage. The star rating for bib shorts was 82% for 5 points, 12% for 4 points, 5% for 3 points, 1% for 2 points, and 0% for 1 point, indicating that 80% or more were very satisfied. Bib tights showed 68% of 5 points, 12% of 4 points, 7% of 3 points, 0% of 2 points, and 1% of 1 point, indicating that 60% and more were very satisfied. The shorts were 5 points 76%, 4 points 18%, 3 points 5%, 2 points 1%, and 1 point 1%, and the tights were 5 points 74%, 4 points 19%, 3 points 6%, 2 points 1%, 1 point was 0%, and it was evaluated that shorts and tights were also very satisfied with more than 70%.

As described above, most of the cycling pants were evaluated as satisfactory, and among them, bib shorts were found to be the most satisfied. However, consumers tend to rate more than 4 out of 5 when evaluating by star rating, and it is necessary to analyze reviews written by consumers themselves.

### Online reviews on cycling pants

#### Positive and negative keywords of the reviews

The online reviews for cycling pants were divided into positive and negative reviews based on their rating. Positive reviews were 6797 documents rated at 4 and 5 points, and negative reviews were 446 documents at 1, 2, and 3. After extracting nouns, adjectives, and verbs by morpheme analysis using KoLNP, the frequencies of occurrence were calculated, and 15 top keywords of the positive and negative are shown in Table 3.

**Table 3:** Word frequency of reviews on cycling pants from 2017 to 2020

Positive review			Negative review		
Word	Frequency	Percent (%)^a^	Word	Frequency	Percent (%)^b^
Size	1130	16.63	Pad	65	14.57
Cost-effective	1011	14.88	Size	64	14.35
Satisfaction	758	11.16	Price	40	8.97
Price	749	11.02	Hip	32	7.17
Pad	610	8.98	Tight	18	4.04
Quality	452	6.65	Thigh	15	3.36
Comfortable	440	6.48	Quality	14	3.14
Reasonable	313	4.61	Textile	12	2.69
Wearing sensation	313	4.61	Bad	11	2.47
Hip	292	4.30	Uncomfortable	11	2.47
Bib shorts	259	3.81	Tighten	10	2.24
Textile	217	3.19	Painful	9	2.02
Tight	211	3.11	Cheap	9	2.02
Good	187	2.75	Thick	8	1.79
Thigh	177	2.60	Band	8	1.79

^a^It was calculated that the word frequency divided by the number of reviews with positive ratings (6797)

^b^It was calculated that the word frequency divided by the number of reviews with negative ratings (446)

In positive reviews, ‘size’ accounted for 1130 cases (16.63%), ‘cost-effective’ with 1011 cases (14.88%), ‘satisfaction’ with 758 cases (11.16%), and ‘price’ with 749 cases (11.02%). In addition, ‘pad,’ ‘quality,’ ‘comfortable,’ and ‘wearing sensation’ appeared. In such positive reviews, the price factor-related price such as ‘cost-effective’ and ‘price’ showed more than 35% of the total positive reviews, so that it is considered to be the biggest important factor in positively evaluating the cycling pants.

In negative reviews, ‘pad’ was 65 cases with 14.57%, ‘size’ with 64 cases (14.35%), ‘price’ with 40 cases (8.97%), ‘cost-effective’ with 33 cases (7.40%), and ‘hip’ with 32 (7.17%). In addition, there were also ‘tight’, ‘quality’, ‘bad’, ‘painful’, and ‘thick’. In negative reviews, ‘pad’ and ‘size’ were extracted as the most important keyword because of accounting for about 30% of negative reviews. Especially, the ‘size’ was derived as the most important keywords not only in negative reviews but also in positive reviews.

**Fig. 5:**
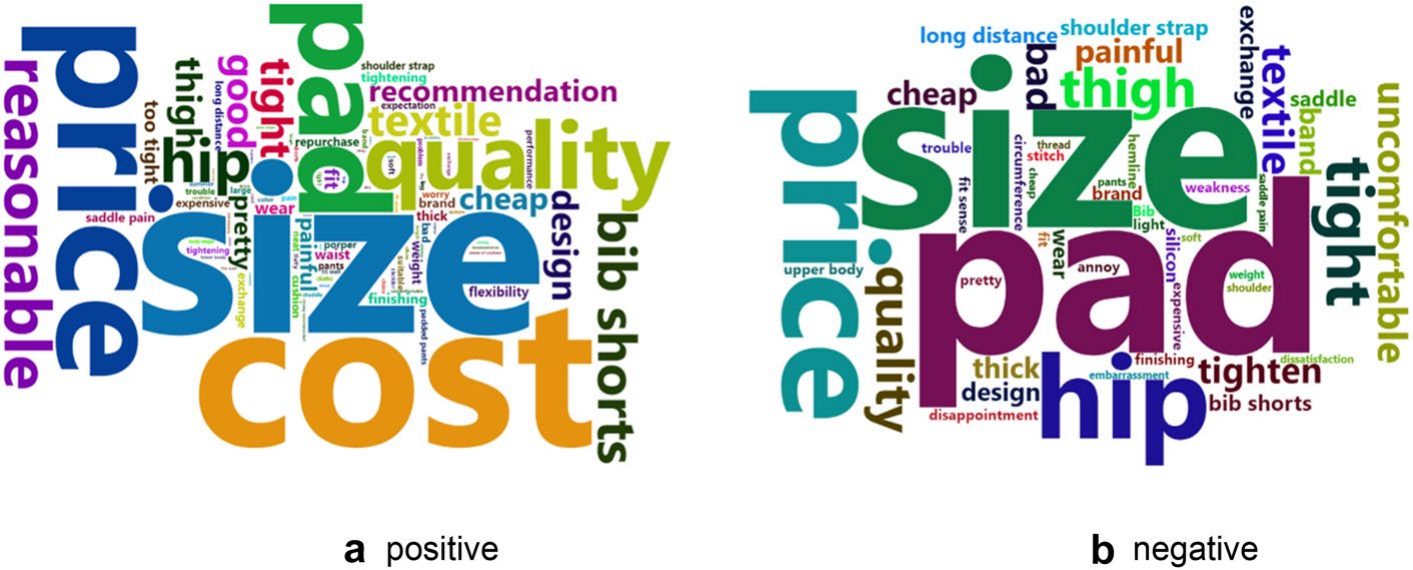
Word clouds of reviews on cycling pants from 2017 to 2020

In addition, the result of visualizing in a word cloud for positive and negative reviews is shown Fig. 5. Just as research on analyzing Internet bicycle cafes (Kim & Yi, 2018), ‘price,’ ‘size,’ and ‘saddle pain’ appeared as the main keywords, the major keywords that affected consumers’ sensibility and post-purchase evaluation of cycling pants were ‘size,’ ‘cost-effective,’ and ‘pad’.

The results of the chi-square test (χ^2^) are shown in Table 4 to determine whether there are differences between bib style (bib shorts and bib tights) and tight style (shorts and tights) in positive or negative reviews. In the bib style, 4061 cases were positive reviews, 56.1%, and 244 cases were negative reviews, 3.4%. In tights style, 2734 cases were positive, 37.8%, and 202 cases were negative reviews, 2.7%. As such, over 90% of all cycling pants were evaluated positively. It was found that there was a difference between positive and negative evaluations depending on whether the shoulder strap was attached (χ^2^ = 4.44, p < 0.05). The bib shorts and bib tights in the suspender style were evaluated more positively than the shorts or tights.

**Table 4:** Differences of the rating of reviews on cycling pants

Cycling pants	Rating		Total n (%)	χ^2^
	Positive n (%)	Negative n (%)		
Bib shorts & bib tights	4061 (56.1%)	244 (3.4%)	4305 (59.5%)	4.44^*^
Shorts and tights	2734 (37.8%)	202 (2.7%)	2936 (40.5%)	
Total	6795 (93.9%)	446 (6.2%)	7241 (100%)	

^*^ p < 0.05

#### Co-occurrence network in positive and negative reviews

Co-occurrence analysis calculates the frequency at which pairs of words appear simultaneously in a document and analyzes them based on the similarity relationship between them (Lee, 2012). The top 300 words in the term-document matrix on positive and negative reviews on cycling pants were converted to a co-occurrence matrix, and the number of simultaneous occurrences between words up to the top 25 among them is shown in Tables 5 and 6.

**Table 5:** Co-occurrence matrix of term matrix in the corpus of positive reviews on cycling pants from 2017 to 2020

	Cost-effective	Size	Satisfaction	Price	Pad	Comfortable	Quality	Reasonable	Hip	Tight	Wear sensation	Textile	Good
Cost-effective	975	119	73	85	92	48	50	42	28	32	26	37	28
Size	119	971	92	97	103	55	75	63	50	88	34	49	25
Satisfaction	73	92	728	125	72	25	75	23	18	18	28	33	9
Price	85	97	125	698	86	39	146	58	28	18	25	35	22
Pad	92	103	72	86	544	58	40	45	79	33	31	50	23
Comfortable	48	55	25	39	58	432	14	7	25	15	21	18	13
Quality	50	75	75	146	40	14	393	35	8	14	15	15	22
Reasonable	42	63	23	58	45	7	35	290	16	28	18	19	1
Hip	28	50	18	28	79	25	8	16	258	16	11	10	2
Tight	32	88	18	18	33	15	14	28	16	223	4	19	7
Wear sensation	26	34	28	25	31	21	15	18	11	4	221	18	16
Textile	37	49	33	35	50	18	15	19	10	19	18	205	9
Good	28	25	9	22	23	13	22	1	2	7	16	9	188
Riding	35	32	25	29	30	30	14	16	24	6	8	8	5
Design	31	31	30	37	33	12	22	18	10	7	17	27	6
Thigh	21	55	22	17	32	14	8	15	20	27	5	14	3
Cheap	21	20	19	71	22	6	19	7	7	6	5	5	6
Recommendation	43	25	20	20	31	10	9	12	8	8	3	9	0
Larger size	25	52	22	12	7	9	16	11	5	21	2	5	2
Bib short	27	33	13	31	30	14	13	15	9	11	7	10	7
Pretty	9	18	9	8	13	11	3	2	6	5	4	6	2
Too tight	4	14	8	6	12	10	4	4	4	4	2	7	7
Fit	10	24	9	10	12	13	6	5	6	6	4	10	4
Wear	11	12	16	11	13	18	8	9	7	4	8	4	3
Painful	10	9	4	8	18	6	2	5	60	2	2	2	1
Finishing	17	22	9	19	13	3	9	9	1	2	3	13	2

	Riding	Design	Thigh	Cheap	Recommendation	Larger size	Bib short	Pretty	Too tight	Fit	Wear	Painful	Finishing
Cost-effective	35	31	21	21	43	25	27	9	4	10	11	10	17
Size	32	31	55	20	25	52	33	18	14	24	12	9	22
Satisfaction	25	30	22	19	20	22	13	9	8	9	16	4	9
Price	29	37	17	71	20	12	31	8	6	10	11	8	19
Pad	30	33	32	22	31	7	30	13	12	12	13	18	13
Comfortable	30	12	14	6	10	9	14	11	10	13	18	6	3
Quality	14	22	8	19	9	16	13	3	4	6	8	2	9
Reasonable	16	18	15	7	12	11	15	2	4	5	9	5	9
Hip	24	10	20	7	8	5	9	6	4	6	7	60	1
Tight	6	7	27	6	8	21	11	5	4	6	4	2	2
Wear sensation	8	17	5	5	3	2	7	4	2	4	8	2	3
Textile	8	27	14	5	9	5	10	6	7	10	4	2	13
Good	5	6	3	6	0	2	7	2	7	4	3	1	2
Riding	185	11	10	7	10	5	10	1	1	7	16	11	2
Design	11	165	11	5	4	4	5	22	3	3	3	3	6
Thigh	10	11	162	3	4	9	10	3	6	1	2	3	6
Cheap	7	5	3	161	6	3	8	3	2	0	0	2	7
Recommendation	10	4	4	6	156	15	5	3	3	4	4	4	2
Larger size	5	4	9	3	15	139	3	1	1	4	3	1	3
Bib short	10	5	10	8	5	3	123	3	7	1	4	3	3
Pretty	1	22	3	3	3	1	3	119	4	6	1	1	2
Too tight	1	3	6	2	3	1	7	4	95	1	1	1	1
Fit	7	3	1	0	4	4	1	6	1	94	3	1	2
Wear	16	3	2	0	4	3	4	1	1	3	85	5	1
Painful	11	3	3	2	4	1	3	1	1	1	5	81	0
Finishing	2	6	6	7	2	3	3	2	1	2	1	0	66

**Table 6:** Co-occurrence matrix of term matrix in the corpus of negative reviews on cycling pants from 2017 to 2020

		Pad	Size	Price	Hip	Tight	Thigh	Quality	Textile	Bad	Uncomfortable	Tighten	Painful
Pad		65	11	8	13	2	6	1	3	3	6	5	3
Size		11	59	4	5	6	6	5	3	3	4	4	0
Price		8	4	40	2	0	3	5	1	2	5	1	0
Hip		13	5	2	32	1	1	0	1	1	1	2	8
Tight		2	6	0	1	18	3	1	1	2	1	4	0
Thigh		6	6	3	1	3	15	1	1	1	2	4	0
Quality		1	5	5	0	1	1	14	0	2	1	1	0
Textile		3	3	1	1	1	1	0	12	0	1	1	0
Bad		3	3	2	1	2	1	2	0	11	0	1	0
Uncomfortable		6	4	5	1	1	2	1	1	0	11	2	0
Tighten		5	4	1	2	4	4	1	1	1	2	10	0
Painful		3	0	0	8	0	0	0	0	0	0	0	9
Rolling up		6	3	2	1	2	3	0	1	2	1	2	0
Cheap		2	3	3	0	1	1	0	1	2	0	1	0
Thick		4	3	0	3	0	1	0	1	0	1	0	0
Band		1	2	0	0	3	2	1	1	0	0	4	0
Exchange		0	3	1	0	1	0	1	0	0	0	0	0
Design		2	0	1	2	0	0	0	1	0	0	0	1
Fall off		2	2	1	0	0	1	0	1	0	1	0	0
Larger size		1	6	0	1	1	2	0	0	0	0	0	0
Saddle		1	0	2	0	1	2	0	2	0	0	1	0
Shoulder strap		1	1	1	0	1	1	1	1	0	0	2	0
Long distance		3	2	0	0	0	2	1	0	1	0	0	1
Rip		1	0	0	1	0	1	0	0	0	0	0	0
Brand		4	4	2	1	1	2	0	1	1	1	1	0

		Rolling up	Cheap	Thick	Band	Exchange	Design	Depreciate	Larger size	Saddle	Shoulder strap	Long distance	Rip	Brand
Pad		6	2	4	1	0	2	2	1	1	1	3	1	4
Size		3	3	3	2	3	0	2	6	0	1	2	0	4
Price		2	3	0	0	1	1	1	0	2	1	0	0	2
Hip		1	0	3	0	0	2	0	1	0	0	0	1	1
Tight		2	1	0	3	1	0	0	1	1	1	0	0	1
Thigh		3	1	1	2	0	0	1	2	2	1	2	1	2
Quality		0	0	0	1	1	0	0	0	0	1	1	0	0
Textile		1	1	1	1	0	1	1	0	2	1	0	0	1
Bad		2	2	0	0	0	0	0	0	0	0	1	0	1
Uncomfortable		1	0	1	0	0	0	1	0	0	0	0	0	1
Tighten		2	1	0	4	0	0	0	0	1	2	0	0	1
Painful		0	0	0	0	0	1	0	0	0	0	1	0	0
Rolling up		9	2	0	1	0	0	1	0	1	0	1	0	1
Cheap		2	9	0	1	0	0	0	1	1	0	2	0	1
Thick		0	0	8	0	0	0	0	1	0	0	0	0	0
Band		1	1	0	8	0	0	0	0	1	1	0	0	0
Exchange		0	0	0	0	7	0	0	1	0	0	0	0	0
Design		0	0	0	0	0	7	0	0	0	1	0	0	0
Fall off		1	0	0	0	0	0	7	0	0	0	0	0	1
Larger size		0	1	1	0	1	0	0	7	0	0	1	0	0
Saddle		1	1	0	1	0	0	0	0	6	1	0	0	0
Shoulder strap		0	0	0	1	0	1	0	0	1	6	0	0	0
Long distance		1	2	0	0	0	0	0	1	0	0	6	0	0
Rip		0	0	0	0	0	0	0	0	0	0	0	6	0
Brand		1	1	0	0	0	0	1	0	0	0	0	0	5

In positive reviews, ‘cost-effective’ was found to have a high frequency of simultaneous occurrences with ‘size,’ ‘pad,’ ‘price,’ ‘satisfaction,’ and ‘comfort.’ ‘Size’ frequently appeared at the same time as ‘pad,’ ‘tight**,’** ‘one size,’ and ‘satisfaction.’ ‘Satisfaction’ also showed at the same time with ‘price,’ ‘cost-effective,’ ‘size,’ ‘pad,’ and ‘quality.’ ‘Satisfaction’ also showed at the same time with ‘price,’ ‘cost-effective,’ ‘size,’ ‘pad,’ and ‘quality,’ and ‘price’ occurred with ‘satisfaction’ and ‘reasonable,’ and ‘price’ with ‘satisfaction’ and ‘reasonable.’ As such, it was found that they were satisfied in terms of cost-effectiveness and generally comfortable to wear. Specific examples of positive are so followed.

“I purchased it because the cost-effective was good. I choose the size based on the size chart, and it was perfect for me. It’s black, so I can wear it with no pressure. The shoulder band is flexible and tighten, and the back is made of mesh, so you could enjoy a cool riding.” (Rating:5, kero****, May 9, 2020)

“I ordered one size larger, and it fits well. The cost-effective is excellent.” (Rating: 4, blue**, Jun 17, 2020)

“As expected, it’s a good for cost-effective. It’s flexible and tight, thin, and light. The pad also has a good cushioning feeling ~ ^_^” (Rating: 5, ekdv****, April 16, 2020)

“Height 174, weight 74, XL fits well. I considered a lot about the L size, but the XL was fitted for me. Delivery was fast, and the quality was better than I thought. It was cost-effective.” (Rating:5, s*a*****, June 23, 2020)

“I personally felt why people are saying “bib short, short.” I could not be more comfortable like this. I was worried because the pads were thin and barely in the front, but when I sat on the saddle, it was rather comfortable without any discomfort. The downside is that I only wore it once and rinsed it with cold water when taking a shower, and after putting it in a laundry net, it was dehydrated the washing machine for a minute, but when I wore it again a day later, the reflective tape of both outer thighs is about to fail off. I think such details are lacking a little bit. The rest are very satisfied!! 173/89, the lower body is thick, but the L size fits perfectly, so it is comfortable.” (Review: 5, jerr****, Jun 21, 2020)

In the positive reviews like above, there are reviews that size fits well, but there were also reviews that it was difficult to select a size such as “I ordered one size larger…” or “I considered a lot about the size,” even though their ratings were good. These reasons are because each brand has a different size system. Therefore, it is considered that an intuitive method for size display is required that allows consumers to select size when purchasing it online easily. In addition, ‘pad,’ ‘satisfaction,’ and ‘comfort’ were showed as keywords, indicating that they prefer cycling pants with pads that are comfortable to wear while reducing saddle pain.

In the negative reviews, ‘pad,’ which had the highest frequency of appearance, showed a high frequency of co-occurrence frequency with ‘hip,’ ‘price,’ ‘uncomfortable,’ and ‘bad.’ That indicates that there were many complaints about the pad. Specific examples of negative reviews are as follows.

“It’s not good. It was the right size for me, but it is uncomfortable because it is a little remain on the hip, too tight in my body, and flowing down the shoulder strap. It’s not recommended.” (Rating: 2, msko****, April 11, 2018)

“Um… I’ve seen a lot of reviews that the cost-effective was good, but it was just mediocre. My height and weight are 180/83. They told me to order a smaller size, so I did a size L, but it was not tight; the back of the pad on the hip is floating a little bit. I don’t know about perfect fitting because it was my first time wearing bib shorts, but it was more ordinary than I thought, and the pain relief was just like that. Still, considering the price, it is good to wear.” (Rating: 3, bulo****, Oct 26, 2017)

“Honestly, my butts hurt. It’s not cost-effective… T_T” (Rating: 2, qhrm**, Jun 2, 2020)

“My hip hurts when I ride longer than 100 km. I had a hard time because the end of the pad touched my butts and got a blister…” (Rating: 3, chul****, Oct 23, 2019)

“Even if I use it for just one month, the fibers on the buttocks are stretched, and I hate to see them. The fibers in the rubber band burst and stretched…” (Rating: 2, as86****, Jun 17, 2020)

As described above, the negative reviews were showed dissatisfaction with the size, pad, and quality, such as “a little remain on the hip,” “hips hurt,” and “the rubber bands burst and stretched.” Recently, as the result of the recent product quality test for cycling pants on the market (Bike Shorts, 2020), the washing resistance satisfied the standards of the recommended quality for textile products, but their durability and friction fastness were found to be insufficient and also most of the products had not antibacterial ability. In addition, the quality of the pad performance evaluation also differed depending on the products, and there were differences in performance between products such as pad thickness and compression hardness. All products were found to be inadequate to the labeling criteria.

As described above, it was found that the quality of the cycling pants was actually insufficient, but the ratings in online shopping malls mainly were good. This indicated that there were the large gabs between the product actual quality and their rating, so these is a problem for consumers to grasp information about the product. For this reason, the reviews are more important than their rating. In addition, consumers trusted the negative reviews more than the positive or the neutral reviews and it influenced purchase intention changes (Son & Rhee, 2007). Thus, although the negative reviews were a few for cycling pants, it seems necessary to focus on negative reviews that will have giving a more useful information of the products.

#### Network analysis of the reviews of the cycling pants

The results of the network analysis of the cycling pants are shown in Table 7. In network analysis, centrality is the concept of influence power, and by analyzing centrality in the cycling pants, it is possible to understand how much each keyword affects the review of the cycling pants.

**Table 7:** Network analysis according to co-occurrence matrix

	Degree	Weighted degree	Modularity class	Eigenvector centrality	Closeness centrality	Harmonic closeness centrality	Betweenness centrality	Clustering
Size	46	4142	0	1.000000	1.00000	1.00000	54.532621	0.610358
Cost-effective	46	2704	1	1.000000	1.00000	1.00000	54.532621	0.610358
Pad	46	3214	0	1.000000	1.00000	1.00000	54.532621	0.610358
Design	45	1082	2	0.987051	0.987952	0.993902	53.549627	0.609259
Satisfaction	46	2216	1	0.990907	0.987952	0.993902	52.489755	0.614198
Riding	46	1484	0	0.982161	0.976190	0.987805	50.360637	0.619304
Price	46	3108	1	0.984771	0.976190	0.987805	49.117675	0.623101
Reasonable	46	1462	0	0.978065	0.964706	0.981707	46.212339	0.631613
Hip	46	1480	0	0.955495	0.942529	0.969512	44.194581	0.635338
Bib shorts	46	1560	0	0.958848	0.942529	0.969512	42.463263	0.640465
Quality	45	1870	1	0.948203	0.931818	0.963415	40.488659	0.642807
Thigh	45	1248	0	0.939688	0.921348	0.957317	39.082163	0.649009
Wear sensation	45	1238	2	0.926739	0.911111	0.95122	38.968275	0.646797
Comfortable	45	1442	0	0.928498	0.911111	0.95122	38.25599	0.65050
Textile	46	1310	2	0.921686	0.901099	0.945122	35.399147	0.660578
Tight	45	1134	0	0.910350	0.881720	0.932927	29.512977	0.683702
Fit	41	470	0	0.819819	0.820000	0.890244	25.13363	0.681548
Cheap	44	690	1	0.837534	0.820000	0.890244	20.959151	0.714286
Tightening	43	554	0	0.822585	0.811881	0.884146	21.873491	0.709165
Wear	42	506	0	0.799190	0.788462	0.865854	16.133729	0.742938
Pretty	40	452	2	0.758732	0.773585	0.853659	18.910614	0.718693
Recommendation	40	692	1	0.773007	0.773585	0.853659	15.511666	0.743497
Larger size	38	496	0	0.759113	0.766355	0.847561	15.480298	0.738095
Shoulder strap	40	428	0	0.761878	0.766355	0.847561	15.349643	0.743734
Band	36	362	0	0.736291	0.759259	0.841463	16.671685	0.72013
Brand	37	458	1	0.739758	0.759259	0.841463	15.455791	0.730519
Leg	39	316	0	0.735271	0.752294	0.835366	16.200435	0.720779
Trouble	39	308	0	0.729570	0.752294	0.835366	15.389813	0.738721
Too tight	37	342	2	0.734017	0.752294	0.835366	14.340071	0.742088
Expensive	37	370	1	0.714261	0.745455	0.829268	14.849385	0.725367
Good	37	584	2	0.720353	0.745455	0.829268	13.755159	0.744235
Thick	39	390	0	0.729403	0.745455	0.829268	12.512108	0.751922
Long distance	41	424	0	0.729776	0.745455	0.829268	12.106226	0.761006
Painful	40	562	0	0.731684	0.745455	0.829268	12.043333	0.765898
Shoulder	41	322	0	0.739435	0.745455	0.829268	10.237288	0.786862
Finishing	38	476	1	0.725068	0.738739	0.823171	9.932602	0.785922
Saddle pain	39	308	0	0.690175	0.725664	0.810976	11.466317	0.767059
Flexibility	36	312	2	0.670906	0.719298	0.804878	11.644361	0.757551
Cushion	35	278	0	0.662886	0.713043	0.798780	11.222881	0.765306
Repurchase	36	276	2	0.661161	0.713043	0.798780	10.874844	0.758503
Bad	35	274	1	0.654937	0.706897	0.792683	9.909139	0.771277
Problem	34	298	0	0.638185	0.700855	0.786585	8.502145	0.774283
Worry	37	272	0	0.647159	0.700855	0.786585	8.492142	0.791859
Rolling up	34	294	0	0.632823	0.694915	0.780488	7.673494	0.793237
Saddle	37	210	0	0.651241	0.694915	0.780488	5.286996	0.84058
Padded pants	0	224	0	0.624102	0.689076	0.774390	6.614893	0.806061
Proper	0	260	0	0.629603	0.689076	0.774390	6.224595	0.814141
Exchange	0	288	0	0.632742	0.689076	0.774390	6.067059	0.826263
Pain	0	268	0	0.604353	0.677686	0.762195	5.274922	0.82835
Clean	0	184	2	0.565124	0.672131	0.756098	8.863233	0.751452
Get down	0	180	0	0.582417	0.666667	0.750000	4.290358	0.843902
Dry	0	162	0	0.560919	0.661290	0.743902	4.851423	0.820513
Performance	0	250	1	0.56714	0.661290	0.743902	4.829425	0.815385
Hem	0	156	0	0.50928	0.650794	0.731707	6.007502	0.763869
Excellent	0	194	1	0.536907	0.650794	0.731707	4.154647	0.832148
Jam	0	122	0	0.536431	0.650794	0.731707	3.842050	0.832148
Flow down	0	160	0	0.507412	0.645669	0.725610	5.020896	0.803303
Length	0	124	0	0.527177	0.645669	0.725610	3.829907	0.833333
Soft	0	198	2	0.527490	0.645669	0.725610	3.679773	0.839339
Uncomfortable	0	146	0	0.525338	0.645669	0.725610	3.678118	0.839339

Eigenvector centrality is useful in finding the most influential central node in the network, and ‘size (1.000),’ ‘cost performance (1.000),’ and ‘pad (1.000)’ are the most influential keywords in the cycling pants. Just as ‘size,’ ‘cost-effective,’ and ‘pad’ were mentioned above as important keywords for both positive and negative, they have proven to be very influential keywords for cycling pants. Next came ‘satisfaction (0.991),’ ‘design (0.987),’ ‘price (0.985),’ ‘riding (0.982),’ ‘reasonable (0,978),’ and ‘bib Shorts (0.959).’

Closeness centrality indicates how close one node is to another node, and the smaller the sum of the connection distances from one node to other nodes in the network, the higher the closeness centrality of the node (Lee, 2012). In closeness centrality, ‘size (1.000)’, ‘cost-effective (1.000)’, and ‘pad (1.000)’ have the shortest connection distance indicating high closeness centrality. Next, ‘satisfaction (0.988)’, ‘design (0.988)’, ‘price (0.976)’, ‘reasonable (0.965)’, ‘bib shorts (0.943)’, ‘hip (0.943)’, ‘quality (0.932)’ were found to have a close connection distance.

Betweenness centrality is a concept that measures how much one node plays the role of an intermediary or bridge in building a network and is used when considering the role of an intermediary (Lee, 2012). As for the betweenness centrality between keywords for cycling pants, ‘size (54.533),’ ‘cost-effective (54.533),’ and ‘pad (53.533)’ were the largest, followed by ‘design (53.550),’ ‘satisfaction (52.490),’ ‘riding (50.361),’ and ‘price (49.118).’ Unlike the closeness centrality and the eigenvector centrality, ‘design’ showed a large value in the betweenness centrality. Keywords with high betweenness centrality can have a great influence in controlling the flow of reviews, so the design can be considered an important factor when purchasing cycling pants. As above, ‘size,’ ‘cost-effective,’ and ‘pad’ had the largest values in eigenvector centrality, closeness centrality and betweenness centrality, and were the most influential central keywords.

**Fig. 6:**
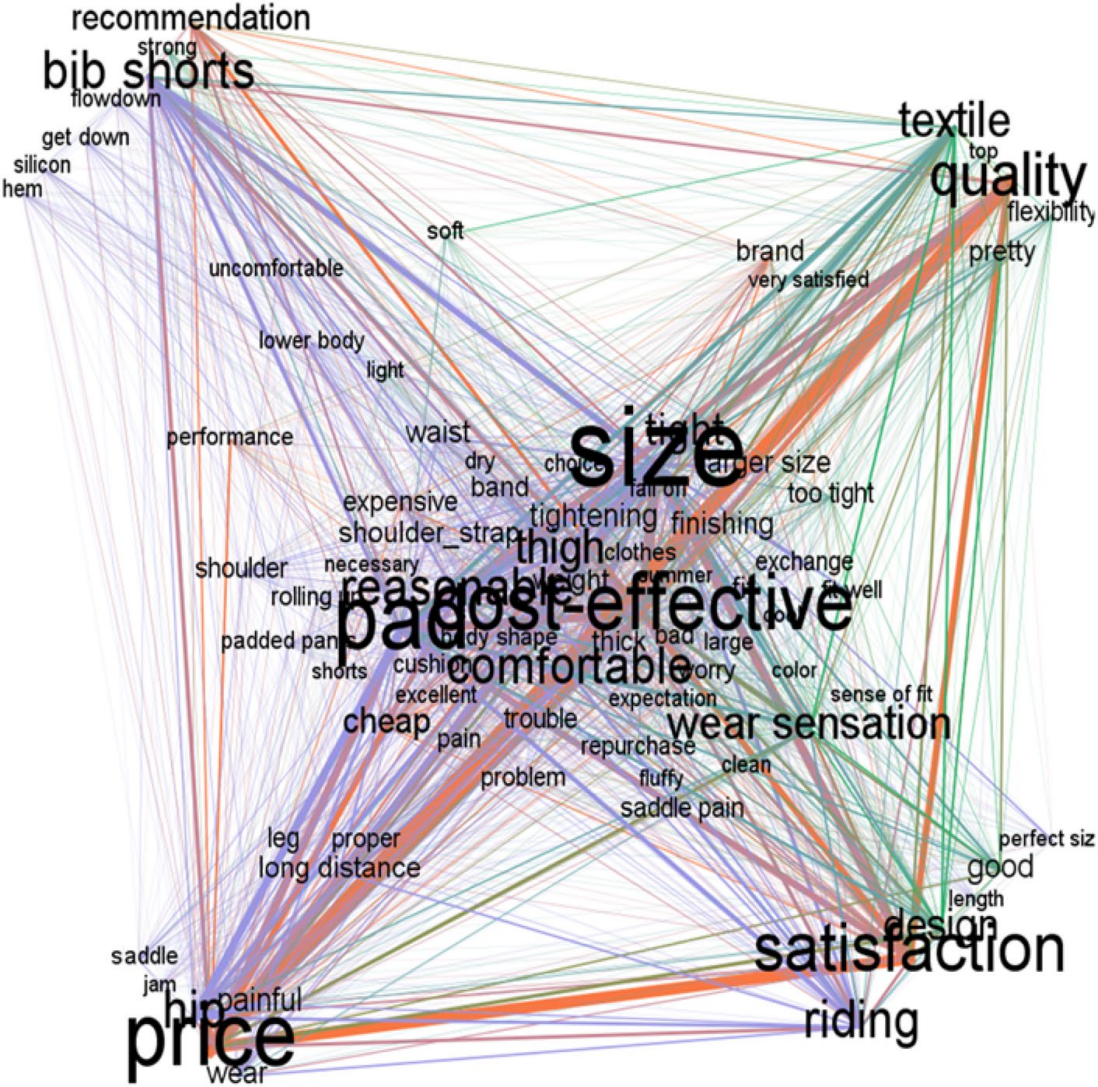
Visualization of network analysis using co-occurrence matrix

The visualization of the network analysis results is shown in Fig. 6. In the cycling pants, ‘size,’ ‘cost-effective,’ and ‘pad,’ which have the largest values in eigenvector centrality, closeness centrality, and betweenness centrality, are in the center, indicating that they are the most important keywords. Besides, keywords related to saddle pain such as ‘price,’ ‘hip,’ ‘saddle,’ ‘painful,’ and ‘jam’ are located nearby, and properties related to a size such as ‘satisfaction,’ ‘length,’ and ‘perfect size’ were grouped. The properties of materials such as ‘quality,’ ‘textile,’ ‘flexibility,’ ‘pretty,’ and ‘brand’ are located closely, and the properties of types and finishes such as ‘bib shorts,’ ‘flow down,’ ‘silicon,’ and ‘hem’ are located nearby each other.

As described above, the most influential keywords in the network analysis for the review on cycling pants were ‘size,’ ‘cost-effective,’ and ‘pad,’ and these were also the keywords with the highest frequency of appearance, both positive and negative. Therefore, these are the most important factor that consumers consider in purchasing decisions in cycling pants.

## Conclusions

This study has investigated the possibility of identifying consumer’s demands and awareness by analyzing reviews on cycling pants in real-time using text mining in online shopping malls. The analysis results for these are as follows.

First, the annual purchase average of cycling pants increased 39% over the 3 years from 2017 to 2019. In addition, in the first half of 2020, the reviews’ frequency increased by more than double the frequency of appearances compared to the first half of last year. It showed that the demand for cycling pants increased rapidly this year. As such, it is believed that the main factor that increased interest and demand for cycle wear in 2020 was the impact of COVID-19, and which led to a surge in bicycle sales leading to the purchase of cycling pants.

Second, the frequency of appearance by types for reviews on cycling pants was in the order of bib shorts, shorts, tights, and bib tights. Among them, bib shorts accounted for more than 50% and were the most preferred. However, there was no significant difference in the number of reviews among bib tights, shorts, and tights. The cycling pants’ rating was rated as very satisfactory by over 70% in all types, and among them, the bib short was the highest.

Third, as the result of analyzing the reviews on the cycling pants by dividing them into positive and negative reviews, positive reviews appeared more than 15 times more than negative reviews. ‘Size,’ ‘cost-effective,’ and ‘price’ were important keywords in both the positive and the negative reviews, and they were played a major role in consumer's sensibilities and post-purchase evaluation when purchasing cycling pants. Besides, bib shorts and bib tights with suspenders style were evaluated more positively than shorts and tights.

Fourth, it was found that there were difficulties in choosing the size not only in the positive reviews but also in the negative reviews. In addition, ‘pad’ had a high co-occurrence frequency such as ‘price,’ ‘uncomfortable,’ and ‘bad,’ indicating a lot of dissatisfaction with the pad.

Finally, ‘design’ was appeared as an important keyword along with ‘size,’ ‘cost-effective,’ and ‘pad.’ Moreover, ‘price,’ ‘saddle,’ ‘painful,’ ‘jam,’ and ‘flow down’ were also extracted.

As such, the demand for cycling pants is increasing recently, and the most influential keywords in the network analysis are ‘size,’ ‘cost-effective,’ ‘pad,’ etc. In addition, riding a bicycle for a long time was reported as a major mechanism for compressing the perineum (Marcolin et al., 2015), so that pad is the most important factor of cycling pants. Moreover, there have been many complaints about pads in online shopping malls, and specific information on cycling pants is lacking so its improvement is required for helping the right purchase of consumers. A previous study that analyzed consumers' reviews for cycling wear online, reported that the results of text mining and the traditional questionnaire survey method on consumer perception are similar (Kim & Yi, 2018). By analyzing unstructured data in real-time using text mining, it is expected that it will be able to understand the target market and analyze consumer requirements or characteristics. In this way, if it is analyzed online consumer reviews with text mining and use to develop a recommendation system, consumers do not have to visit various websites to search numerous reviews for product information, especially electronic word of mouth. Also, the reason for the need for text mining according to each product line is that each product’s required performance is different, so text mining for each product category will provide more accurate product information to consumers. It is also necessary to have a system that converts them into quantitative data by converting them into a database by identifying synonyms and similar words of various texts that consumers use about cyclewear on the Internet and constructing a text and a stopword dictionary.

However, as a limitation of this study, the scope of crawling is limited to the internet shopping mall, so it is unreasonable to generalize the results. In order to use these as objective indicators in product development, it is necessary to expand the scope of crawlings, such as various portal sites, blogs, Twitter, and Facebook. In addition, the database of reviews and product information for cycling pants is expected to help in the development of a recommendation service system that can make consumption more accurate and smarter for consumers.

## Data Availability

The datasets used and/or analyzed during the current study are available from the corresponding author on reasonable request.
